# Review of Personalized Medicine and Pharmacogenomics of Anti-Cancer Compounds and Natural Products

**DOI:** 10.3390/genes15040468

**Published:** 2024-04-08

**Authors:** Yalan Zhou, Siqi Peng, Huizhen Wang, Xinyin Cai, Qingzhong Wang

**Affiliations:** 1 Institute of Chinese Materia Medica, Shanghai University of Traditional Chinese Medicine, Shanghai 201203, China; zhouyalan66@gmail.com (Y.Z.); pengsiqi9806@gmail.com (S.P.); hamy1314haha@163.com (H.W.); 2 Shanghai R&D Centre for Standardization of Chinese Medicines, Shanghai 202103, China

**Keywords:** personalized therapy, pharmacogenomics, personalized medicine, anticancer, natural products

## Abstract

In recent years, the FDA has approved numerous anti-cancer drugs that are mutation-based for clinical use. These drugs have improved the precision of treatment and reduced adverse effects and side effects. Personalized therapy is a prominent and hot topic of current medicine and also represents the future direction of development. With the continuous advancements in gene sequencing and high-throughput screening, research and development strategies for personalized clinical drugs have developed rapidly. This review elaborates the recent personalized treatment strategies, which include artificial intelligence, multi-omics analysis, chemical proteomics, and computation-aided drug design. These technologies rely on the molecular classification of diseases, the global signaling network within organisms, and new models for all targets, which significantly support the development of personalized medicine. Meanwhile, we summarize chemical drugs, such as lorlatinib, osimertinib, and other natural products, that deliver personalized therapeutic effects based on genetic mutations. This review also highlights potential challenges in interpreting genetic mutations and combining drugs, while providing new ideas for the development of personalized medicine and pharmacogenomics in cancer study.

## 1. Introduction

In recent years, with the completion of the Human Genome Project, the development of genomics, proteomics, and imaging technology and the advent of molecularly targeted drugs, personalized medicine has come into being [1,2,3]. Personalized medicine is an emerging field, in which physicians utilize diagnostic tests to determine which medical treatments will work best for each patient or apply medical interventions to alter molecular mechanisms that impact health. By combining data from diagnostic tests with an individual medical history, circumstances, and values, medical staff can develop targeted treatment and prevention plans with their patients. According to the concept of personalized medicine, the treatment plan is selected and determined based on information about the patient’s individual genetics, environment, and lifestyle. In this way, each patient can achieve the maximum health benefits, while reducing ineffective treatments and side effects [4]. Up to 2018, 355 pharmacogenomic biomarkers and 284 drugs have been approved by the FDA. Those pharmacogenomic biomarkers approved for use in personalized medicines are usually specific genetic variants (i.e., gene tags) or abnormally expressed proteins [5]. They can help distinguish those who will or will not respond to a drug, prevent adverse drug reactions, and play an important role in optimizing drug dosing [6,7,8]. For those personalized medicines, anti-tumor drugs have a larger proportion, which mainly resulted from the discovery of oncogenic driving genes and the development of molecular targeted drugs [9]. These personalized oncology drugs mainly include receptor tyrosine kinase inhibitors, small molecule inhibitors, vaccines, antibodies/small molecule–antibody conjugates, and monoclonal antibodies [10,11,12]. Besides new personalized drugs, repositioning drugs is also an important approach to personalized drug discovery, which mainly involves developing and gene-tagging “complementary diagnostic” biomarkers for drugs already on the market [13,14,15]. In the case of the lung cancer drug ramucirumab, for example, the FDA cautions that patients treated with this drug must be those with epidermal growth factor receptor (EGFR) and ALK receptor tyrosine kinase (ALK) mutations [16,17]. The new use of old drugs can not only save a lot of time and cost while ensuring efficacy, but also reduce safety risks and speed up drug approval [18]. These drugs are mainly used in cancer, neuropsychiatric diseases, infections, cardiovascular diseases, metabolic diseases, and other areas [19,20,21,22,23]. With the advent of increasingly personalized medicine, Big Data analytics is creating a model of precision medicine that allows for a more detailed treatment plan according to the comprehensive situation of a person’s genes, lifestyle, environment, and other factors. This can not only reduce the safety risks of drugs, but also greatly improve their effectiveness [24]. Based on the theme of personalized medicine, this review mainly discusses the strategies of discovering novel drugs, chemical antitumor agents, natural antitumor products, and so on.

## 2. Technical System for the Development of New Drugs and Personalized Medicine

The main goal of personalized medicine is to enable precise treatment with drugs for specific populations and types of diseases through design, screening, and optimization [25,26]. With the continuous innovation and development of life sciences and technologies, the research and development strategies for new drugs have changed in recent years from the traditional models based on a disease phenotype, local signaling pathway, and single target. These new models are based on the molecular typing of the disease, the global signaling network in the organism, and all targets [27,28,29]. They have also greatly promoted the research and development of personalized medicine. The rapid development of new omics technologies, rapid accumulation of multidimensional and large-scale omics data, advent of molecular imaging, advent of supercomputers, and continuous improvement of correlation analysis and mining algorithms have all contributed [30,31,32,33,34,35,36,37]. Next, we will focus on the emerging research in personalized medicine and the development of new key technologies for systematic deployment (See Table 1 for more supplementary information).

### 2.1. Artificial Intelligence Technology

Artificial intelligence is a branch of computer science that aims to simulate human thought processes, learning abilities, and knowledge reserves [38,39]. In recent decades, the improvement of computer speed and the rapid development of artificial intelligence (AI) have promoted the study of drug discovery and personalized medicine [40,41]. As one part of the development of personalized medicine, large-scale biomedical data were being used to identify the biological principles behind drugs to more accurately simulate and predict the complex effects of drug molecules in vivo [42]. In late 2016, Goldman Sachs Group published a comprehensive report on artificial intelligence in the ecology and future of AI. This report pointed out that the application of AI and powerful algorithms will help to get rid of the risks in the development of new drugs [43]. In 2021, one study reported that machine learning was able to predict the higher accuracy equal to 76% for the outcome of phase III clinical trials of anti-tumor agents for the treatment of prostate cancer [44]. In the field of disease diagnosis, a study has shown that melanoma diagnoses using machine learning can reach the level of well-experienced dermatologists [45]. In addition, Google company has developed DeepMind’s Deep Learning algorithm, which can quickly and accurately detect early signs of diseases, such as age-related macular degeneration and diabetic retinopathy, to prevent and treat them in advance [46,47]. Artificial intelligence technology, with its powerful automatic feature extraction, complex model building, and image processing, opens up new possibilities for the analysis, processing, and application of biomedical Big Data [48,49,50].

### 2.2. Approaches with Multidimensional Omics Data

Theoretically, large-scale integrated analysis of multidimensional omics data can provide comprehensive and complete insight into the molecular mechanisms of complex diseases and specific drugs. That is, genome-wide data encompassing the genome, transcriptome, proteome, metabolome, and other various dimensions can help to analyze the target, regulatory mechanism, and biological effect of drugs at different molecular levels [51,52]. In this way, the targeting and off-target effects of the drug can be revealed globally, and different drugs can be combined according to the different characteristics of each drug to improve efficacy and reduce side effects. Thus, personalized and accurate guidance for drug treatment of diseases is achieved [53]. Meanwhile, new potential targets, novel genetic variants, and regulatory mechanisms can be screened out from the genome-wide approaches, which will help to improve the effectiveness and successful probabilities of the new use of old drugs to a certain extent [54,55]. At present, the strategies of personalized medicine discovery based on multi-omics data are not yet mature, but they have been tentatively applied in the phase of personalized medicine discovery [56]. Recent studies have shown that by integrating multi-omics data, including SNP and copy number variants, mRNA expression profiling, and protein profiling of lung adenocarcinoma, normal tissue, and tumor xenograft models. It has been predicted that the changed proteins that cannot been found from the single-omics data are strongly correlated with cell metabolism and survival in patients with lung cancer and other cancer [57,58]. In addition, integration analysis between transcriptomic and proteomic data from 24 human tumor xenograft models of breast cancer patients has shown that proteomics can better detect dynamic changes in some proteins and protein phosphorylation, including AKT and ARAF, BRAF, and HSP90AB1 [59]. All in all, multi-omics integration can provide strong technical support for clinical personalized treatment and personalized medicine development [60].

### 2.3. Study on High-Throughput Targets with Chemical Proteomics Technology

The function of chemical proteomics technology is to exploit the specific interaction between the drug and the target protein. By combining various enrichment methods with high-resolution biomass spectroscopy, the target proteins and molecular regulatory mechanisms can be studied [61]. This method can identify binding targets of small-molecule compounds from complex biological samples (cells or tissues), with the advantages of high throughput and unbiasedness. Chemical proteomics technology can provide important information for further analysis of the full target spectrum of active molecules at the cellular level and evaluation of drug activity, toxicology, and indications [62,63]. Currently, chemical proteomics-based technologies are being used to identify drug targets. These primarily cover affinity-based protein profiling, activity-based protein profiling, thermal proteomic profiling, and affinity-dependent drug target stability [64]. We listed the principles, advantages, and disadvantages of those four technologies.

### 2.4. Computer-Aided Drug Discovery (CADD) System

CADD technology can be used in various phases of the development of new personalized medicine. These comprise drug target identification, lead discovery and optimization, therapeutic marker and predictive model discovery, drug combination and repositioning research, ADME and safety research, etc. [65,66,67,68]. CADD can reduce costs and shorten the time of research and development, while greatly improving the successful risk [69]. We will focus on the application of computational methods in target identification and lead discovery in personalized medicine. (**1**) Target identification: currently, there are still a large number of potential targets to be explored for effective drug development [70]. The potential targets of chemical compounds can be predicted using approaches that incorporate bioinformatics and cheminformatics at multiple levels to improve the reliability of data analysis and effectively promote the use of computers. These include chemical structure similarity searches, data mining using machine learning methods, reverse molecular docking, and algorithm-based bioactivity profiling [71,72,73]. Keiser et al. (2018) developed the similarity ensemble approach to enable large-scale searches for known drug targets by comparing the molecular fingerprint similarities of thousands of compounds [74]. In addition, the dynamic structure and thermodynamic dynamics parameters of molecules and protein can be fully obtained by simulating molecular dynamics, which is helpful in the search for new personalized drug targets [75,76]. (2) Lead compounds discovery: Molecular docking is the most commonly used method for designing drugs based on receptor structure [77]. These related software programs include Gold, AutoDock, GLIDE, etc. [78]. In addition, molecular dynamics (MD) can also be used to study the dynamic structure of proteins, such as AMBER (Amber Molecular Dynamics), GROMACS (GROningen MAchine for Chemical Simulations), and NAMD (NAnoscale Molecular Dynamics) [79]. The free energy of the binding of the protein to the ligand was calculated using MD, and then the binding mode and the binding activity of the two were predicted to screen the lead compounds [80]. For ligand-based drug design methods, compound similarity analysis, quantitative structure–activity relationship (QSAR), and pharmacophore model analysis have been successfully used for lead discovery [81,82]. For example, Mueller et al. (2012) used QSAR to identify 27 allosteric modulators of the mGlu5 receptor that can be used to treat anxiety disorders, Parkinson’s disease, and schizophrenia [83]. In addition, Ijjaali et al. (2007) performed ligand-based virtual screening of 2 million compounds and identified 16 highly active human T-type calcium channel blockers that could be used to treat epilepsy and neuropathic pain [84]. All these approaches have helped to identify the new drug design process, and they are usually combined multiple approaches to design and optimize lead structures.

## 3. Anti-Cancer Personalized Medicine

The main purpose of personalized therapy is to develop effective targeted drugs for different sub-phenotypic patients. The application of personalized anti-cancer drugs has been widespread due to the discovery of oncogenic-driving genetic mutations in the development of molecular targeted drugs. These personalized anticancer drugs mainly include tyrosine kinase inhibitors, small molecule inhibitors, vaccines, antibodies/small molecule antibody conjugates, and monoclonal antibodies [12,85,86]. Among these personalized medicines, a great number of drugs were found from drug repositioning, which identifies new therapeutic opportunities for existing drugs. For example, pembrolizumab, novolumab, and other FDA-approved drugs are used for personalized treatment through drug repositioning [87,88]. We have summarized the FDA-approved personalized anti-tumor drugs (see Table 2 for details). Here, we focused on personalized anti-cancer agents targeting ALK and EFGR receptors.

**Table 1:** Screening and comparison of drug targets based on chemical proteomics.

Technology	Principle	Advantage	Disadvantage
AfBPP	Affinity of target proteins to active small molecules on stationary phases	1. No bias; 2. Systematic study of total protein; 3. It can enrich the target and is suitable for identification of low-abundance proteins.	1. A detailed understanding of the structure–activity relationship of active molecules is required; 2. Chemical derivatization of active molecules is required; 3. Targets with low abundance and low affinity are easy to be washed off; 4. Probes usually cannot enter cells.
ABPP	The target protein forms a covalent bond with a covalent small molecule.	1. No bias; 2. Systematic study of whole protein; 3. It can enrich the target and is suitable for identification of low-abundance proteins; 4. Grasp low-affinity targets; 5. Probes usually get into cells.	1. A thorough understanding of the structure–activity relationship of active molecules is required; 2. Chemical derivatization of active molecules is required; 3. Non-specific covalent binding is easy to occur.
TPP	The thermal stability of the target protein increases after binding with small molecules, and it is not easy to precipitate	1. No bias; 2. Systematic study of whole protein; 3. No derivations of small active molecules are required.	1. Limited effect on extreme conditions, such as heat insensitivity or heat-unstable proteins; 2. Further measures should be taken to reduce the complexity of samples so as to realize the identification of low-abundance proteins.
DARTS	The stability of the target protein increases after binding with small molecules and is not easily degraded by enzymes	1. No bias; 2. Systematic study of whole protein; 3. No derivations of small active molecules are required.	1. The protein that is not sensitive to enzyme digestion has limited effect; 2. Further measures should be taken to reduce the complexity of samples so as to realize the identification of low-abundance proteins.

### 3.1. ALK Inhibitors

Anaplastic lymphoma kinase (ALK) is an important molecular marker of non-small-cell lung cancer [89,90]. In 2007, EML-ALK, the ALK fusion gene, was found to be present in 3%–7% of patients with non-small-cell lung cancer, triggering the research and development boom of ALK personalized medicine [91]. (1) Crizotinib is an orally administered ALK inhibitor that effectively inhibits phosphorylation of the NPMALK fusion protein in human degenerative cell lymphomas Karpas-299 and SU-DHL -1 cells. Cell cycle arrest and apoptosis were significantly increased after 24- and 48-h treatment with crizotinib. In mice transplanted with a Karpas-299 tumor, oral administration of 100 mg/kg crizotinib for 15 days resulted in complete tumor regression. This is consistent with antitumor activity in vivo. (2) Ceritinib is a second-generation ALK inhibitor synthesized by Pierre-Yves Michelly’s team at Novartis based on TAE684. Cell proliferation assays showed that ceritinib significantly inhibited proliferation of Karpas-299 and BaF3 tool cells with high expression of the ALK fusion protein. A 25 mg/kg dose of ceritinib significantly inhibited the growth of subcutaneously transplanted tumors in nude mice with Karpas-299 and NCLH2228 tumor cell lines. Ceritinib has some ability to cross the blood–brain barrier, and the brain-tissue-to-plasma exposure ratio measured by isotopic labeling is approximately 15% [92,93,94]. (3) Alectimib is an orally active and highly selective inhibitor ALK. Cell-level studies have shown that alectimib can inhibit the activation of ALK and downstream STAT3 and AKT signaling pathways in NCL-H2228 non-small-cell lung cancer cells. Meanwhile, alectimib significantly inhibited the proliferation of NCI-H2228 non-small-cell lung cancer cells. In nude mice, alectimib at a dose of 6 mg/kg significantly inhibited the growth of NCI-H2228 and KARPAS-299 cell transplantation tumors. In terms of the ability to overcome mutations, 100 nM alectimib inhibited the phosphorylation of ALK in BaF3/EML4- ALK -L1196M cells [95,96]. (4) Brigatinib is a dual-target inhibitor of ALK and EGFR. Subsequent pharmacodynamic evaluation studies showed that at the molecular level, brigatinib significantly inhibited kinase activity mutated by C1156Y, F1174L, L1196M, G1202R, and R1275Q ALK and ALK, while brigatinib inhibited EGFR and other kinase activities mutated by ROS1, FLT3, and D835Y. At the cellular level, brigatinib was shown to inhibit proliferation and intracellular ALK protein phosphorylation of AlK-driven lymphoma cells Karpas-299, SU-DHL -1, L-82, and SUP -M2 and lung cancer cells H3122 and NCLH2228.The results showed that 50 mg/kg brigatinib could lead to shrinkage or even complete regression of Karpas-299 and NCI-H2228 transplanted tumors in mice. No significant reversal of tumor growth inhibition was observed 15 to 30 days after drug discontinuation. A 50 mg/kg dose of brigatinib significantly inhibited intracranial lesions and prolonged survival in mouse models of brain metastases containing transplanted NCLH2228 tumors. In mice with BaF3/EML4- ALK -G1202R grafts, 25 mg/kg and 50 mg/kg brigatinib inhibited tumors 55% and 88%, respectively [92,94,97]. (5) Lorlatinib, a third-generation ALK inhibitor, showed significant inhibitory activity against ROS1 and ALK kinases. The tumor transplantation experiment in mice showed that 25 mg/kg lorlatinib could significantly inhibit tumor growth in H1322 and H3122/EM1A-ALKLSH312/EMLA-ALKG1269A mice. Lorlatinib (10 mg/kg) can significantly inhibit transplanted tumor growth in the tissue-derived mouse model of tumor transplantation, and a tumor can also significantly decline when lorlatinib is replaced after crizotinib resistance. In an intracranial mouse transplant tumor model, lorlatinib significantly inhibited internal tumor growth and significantly prolonged mouse survival [98,99,100].

### 3.2. EGFR Inhibitors

EGFR is a member of the epidermal growth factor receptor family of ErbB receptor tyrosine kinases, which also includes BGFR, ErbB2 (HER2), ErbB3(HER3), and ErbB4(HER4) [101,102]. EGFR is a transmembrane receptor protein consisting of extracellular ligand-binding domains, transmembrane domains, and intracellular kinase-active domains [103]. Upon binding of the extracellular ligand-binding region to a ligand, homodimerization or heterodimerization occurs, followed by autophosphorylation in the intracellular region, to activate its kinase. Phosphorylated EGFR terminals bind to various downstream adaptor proteins and perform various physiological functions, such as maintaining cell growth and inhibiting cell apoptosis through movement. EGFR is expressed in a variety of tissue cells [104]. Under normal physiological conditions, EGFR regulates a number of biological processes, such as cell proliferation and differentiation. For example, high expression of EGFR or abnormal activation is associated with the development and progression of various tumors, such as non-small-cell lung cancer (NSCLC), metastatic colorectal cancer (mCRC), head and neck cancer (HNSCC), glioblastoma (GBM), ovarian cancer, and so on. Among these tumors, the occurrence and development of NSCLC are most closely associated with EGFR, and the molecular mechanism of EGFR driving the occurrence and development of NSCLC is also the most profound. Several EGFR inhibitors have been used to treat NSCLC. The discovery of EGFR-activated mutations as sensitive markers for small-molecule EGFR inhibitors is not only a milestone in the history of lung cancer treatment, but also a model for personalized tumor treatment [102,103,104,105].

EGFR inhibitors include primarily gefitinib and erlotinib [106]. They bind to the EGFR kinase region in a competitive ATP-binding manner, reversibly inhibiting EGFR kinase activity and thus blocking downstream signaling. (1) Gefitinib was approved by the FDA in 2003 for the treatment of patients with advanced NSCLC who have failed chemotherapy [107]. (2) Erlotinib was approved by the FDA in 2004 for the treatment of locally advanced or metastatic NSCLC and was subsequently approved in combination with gemcitabine for the treatment of locally advanced or metastatic pancreatic cancer [108]. The discovery of the deletion mutation in exon EGFR19 (exon19del) and the base substitution mutation L858R in exon 21 (L858R mutation), a sensitive marker, uncovered the reason why some people are sensitive to EGFR inhibitors. EGFR-sensitive mutations are located in the intracellular ATP-linked pocket kinase region. They increase the affinity between the binding pockets and ATP, leading to disruption of the EGFR self-inhibition pathway and continuous activation of downstream signaling pathways, which in turn causes carcinogenesis. The affinity of gefitinib and erlotinib for this mutant EGFR protein is stronger than that of ATP molecules, leading to more severe clinical effects in patients with these mutations. Therefore, EGFR mutation detection has been approved for first-line clinical treatment of progressive EGFR-mutated NSCLC and has become routine clinical practice in most cancer centers worldwide. Although gefitinib and erlotinib are effective in treating NSCLC with EGFR-sensitive mutations, patients develop drug resistance within an average of 9 to 14 months after treatment, severely limiting the clinical use of first-generation inhibitors [106,109,110,111]. The emergence of resistance mutations and the activation of compensatory signaling pathways are the main causes of drug resistance. The EGFR T790M mutation was the most common cause of drug resistance and accounted for more than 50% of acquired drug resistance. Therefore, the research and development of second-generation EGFR inhibitors targeting EGFR wild-type and EGFRT790M-resistant mutations has attracted much attention. (3) Afatinib, which can covalently bind the EGFR ATP binding site C797 in the pocket, has significant inhibitory activity against both EGFR WT and T79M resistant mutations. The drug was approved in 2013 by the US FDA for the treatment of advanced non-small-cell lung cancer and HER2-positive advanced breast cancer, and in 2016 for the treatment of patients with advanced lung cancer, whose disease has progressed after platinum-based chemotherapy [112]. However, afatinib has resulted in significant side effects due to its potent inhibition of wild-type EGER activity and has been unable to achieve the effective blood levels of EGFR T790M in humans. Therefore, the research and development of third-generation EGFR inhibitors that selectively inhibit resistant EGFR T7M mutations has attracted much attention [105,113]. (4) Osimertinib can covalently bind to the cysteine site of EGFR 797 and selectively inhibit EGFR-sensitive and drug-resistant mutations. Osimertinib weakly inhibits EGFR wild-type and clinically shows good efficacy and few side effects in patients with drug-resistant mutations containing EGFR T790M. Osimertinib was approved by the US FDA in November 2015 for the second-line treatment of patients with metastatic NSCLC containing EGFR T790M. In 2018, osimertinib was approved by the US FDA as a first-line treatment for patients with EGFR-sensitive, mutation-positive metastatic NSCLC. In addition, osimertinib can effectively cross the blood–brain barrier and is effective in patients with brain metastases (including meningeal metastases) from lung cancer, which is a major advantage over other small-molecule EGFR inhibitors [114,115,116].

## 4. Pharmacogenetics of the Anti-Cancer Natural Products

The action of drugs on the body is generally divided into two phases, the pharmacokinetic phase and the pharmacodynamic phase, and the actual action of drugs in vivo begins with binding to targets; therefore, the pharmacodynamic phase cannot be ignored [117]. It is caused by targeting specific molecular mechanisms and signaling pathways, and at the same time, drug–target interactions are influenced by genetic variations in genes [118,119]. Numerous bioactive natural products have been discovered and isolated, but the role of genetic variations in targets has not been adequately explored. Moreover, few pharmacological targets have been clearly confirmed in comparison with metabolizing enzymes and transporters [120,121]. It is reported that in people with different pharmacological target genotypes, the drug effects of natural products differ. Some genetic variations affect drug action in other ways, such as modulating the functions of related proteins that are not the direct target proteins, enzymes, or transporters [122]. As a result, the situation is more complex, and more attention needs to be paid to drug-related pathways. For some natural products, there are defined targets, but there are still a large number of natural products that are linked not only to their direct targets, but also to other indirect reactions. However, evidence of direct interactions has been found to be difficult to obtain, making the study of the molecular targets of natural products very challenging. The interactions between natural products and target sites are mainly described in the following examples using tumors (see details in Table 3).

Currently, genome-wide association studies (GWASs) and other techniques have found many compounds with anti-tumor activities, including many natural products with anti-tumor activities through various mechanisms, such as cytotoxicity [123,124]. This biological action leads to a variety of biological responses, such as inhibition of mitosis, DNA damage, DNA synthesis, and repair damage [125]. The cytotoxic pathways may be related to the efficacy and adverse effects of natural tumor products.

Trabectedin is a marine-derived natural product originally isolated from the marine ascidian *Ecteinascidia turbinate*. Trabectedin has a complex mechanism of action that affects important cell biological processes in tumor cells and is the first marine anticancer agent approved for patients with soft tissue sarcoma (STS) in the European Union [126]. The DNA damage induced by the drug is largely caused by the nucleotide excision repair protein (NER) and an arginine residue in Rad13 (Arg961). Trabectedin can kill cells by poisoning the DNA NER machinery and DNA repair pathways of homologous recombination [126]. Trabectedin has a unique, multilayered mechanism involving transcriptional regulation and DNA repair systems. In addition, transcription-coupled nucleotide excision repair and homologous recombination repair (HRR) are the main features of its antiproliferative activity. It also has the ability to modulate the tumor microenvironment, which can alter the function and expression of DNA repair genes, such as BRCA1 and BRCA2 [127,128]. BRCA proteins play a critical role in DNA repair, as they are essential for the repair of double-strand breaks. Cancers that have a mutation in the BRCA1 or BRCA2 genes that reduce protein activity, as in ovarian and breast cancers, may increase the activity of drugs that exert their cytotoxicity via DNA double-strand breaks [129,130]. As for BRCA2 mutations, trabectedin showed higher antitumor activity in relapsed metastatic breast cancer patients with germline BRCA2 mutations than in those with BRCA1 mutations. Loss of the wild-type BRCA2 allele in the tumor results in an excellent early, complete metabolic response due to a somatic aberration that likely leads to deregulation of the cellular HR function responsible for increased sensitivity to trabectedin [128]. These reports demonstrate the importance of BRCA1/2 mutations in the administration of trabectedin for the treatment of tumors with defective DNA damage repair.

Vincristine is isolated from the plant *Catharanthus* roseus and is a drug widely used in cancer treatment. It can be used to treat leukemias, lymphomas, brain tumors, and also solid tumors [131]. The mechanism of tumor restriction is due to its interference with microtubules in the mitotic spindle [132]. In the pharmacokinetics of vincristine, CYP3A enzymes and ABC transporters may play an important role. As with other substrates of CYP3A enzymes, genetic variants of CYP3A also lead to its adverse effects, such as vincristine-induced peripheral neuropathy [133]. There are studies confirming that active CYP3A5 expressors have a lower risk of VIPN than nonexpressers [134,135]. However, a lower risk of VIPN has been observed in some children with the CYP3A5*3 genotype. This is an inverse conclusion when toxicity is accumulated to the highest concentration without CYP3A5 enzyme activity [136]. We need to pay more attention to the neurotoxicity caused by genetic variations of vincristine and perform clinical optimization of its metabolism.

Gigantol, a bibenzyl phenolic compound derived from several medicinal orchids, has been shown to inhibit proliferation, migration, EMT, and the cancer stem cell (CSC) phenotype in lung cancer cells [137,138,139]. At non-toxic doses (below 20 μM), gigantol isolated from *Dendrobium draconis* could suppress tumor spheroid formation and decrease lung CSC marker proteins, including CD133 and ALDH1A1, in non-small-cell lung cancer NCI-H460 cells. Additionally, gigantol inhibited cancer stem-cell-like phenotypes through down-regulation of the AKT signaling pathway, which leads to reduced levels of Oct4 and Nanog [137].

Paclitaxel (PTX) is one of the natural broad-spectrum antitumor drugs used as first-line chemotherapy in ovarian cancer therapy [140]. The efficacy of paclitaxel is associated with ABCB1 G2677T/A mutation. ABCB1, also known as MDR1, is the efflux pump of cells. After paclitaxel enters human tumor cells, it is pumped out of the cells by ABCB1 [141]. The mutation at position 2677 reduces the transport capacity of ABCB1, allowing the drug to accumulate in tumor cells and achieve a good therapeutic effect. Studies have shown that in patients with ovarian cancer, the G2677T/A mutation has a good effect on paclitaxel [142].

Chrysotoxine, a bibenzyl compound isolated from stems of *Dendrobium pulchellum*, has been reported to sensitize anoikis and inhibit metastasis of lung cancer cells in an anchorage-independent fashion. Bhummaphan et al. (2019) investigated the suppressive effect of chrysotoxin on CSC-rich populations of H460 and H23 cells and primary CSCs in three-dimensional (3D) cultures. The result showed that non-toxic doses (≤20 μM) of chrysotoxin inhibited CSC-like phenotypes and decreased CSC markers CD133, CD44, ABCG2, and ALDH1A1, which was mediated by a Src-AKT-Sox2-dependent mechanism [143].

## 5. The Challenges of Personalized Therapy

Although a significant number of gene variants’ specific anti-tumor drugs have been approved by the FDA, the percentage of personalized medicines is less than 10% of all the FDA-approved drugs [144]. The development of personalized medicines still confronts some challenges: (1) Interpretation of genetic variations: thousands of mutant genes are scattered throughout the genome of cancer cells, and there are hundreds of different mutant genes associated with cancer [145]. According to current clinical statistical data on personalized tumor treatment, only 30–50% of patients can link the tumor to the corresponding mutations. Moreover, only 3–13% of patients can choose personalized therapy through individual genomic analysis [146]. At present, genome sequencing technology, especially single-cell sequencing and omics analysis, has been rapidly developed. We are able to obtain whole genome data in a relatively short time and at low cost [147,148,149]. Therefore, people need to not only interpret gene functions, but also decipher the functional effects of gene mutations. (2) Dynamic molecular changes of cancer: the pathogenic genes in cancer patients can usually evolve and escape the therapeutic effect of drugs on the lesion through genetic mutation, which is referred to as secondary drug resistance. After secondary drug resistance, current diagnostic and treatment methods may no longer be applicable in the original disease state. Due to our limited understanding of complex signal transduction pathways, the development of personalized treatment options based on basic research cannot resolve the new disease molecular state with some variants and mutants [150]. Therefore, we have to confront significant challenges in monitoring the molecular typing of patients, identifying and defining the occurrence of drug resistance, and finding new treatment options for the protective mechanism of tumors. (3) Combination of drugs: In developing personalized treatment, it is rare for a single drug to act on all the gene mutations causing a patient’s disease. Therefore, it is more beneficial to use a combination drug to treat diseases with multiple genes [151]. Currently, drug combinations are commonly used for cancer, infectious diseases, cardiovascular diseases, and other areas to obtain reasonable strategies for the development of drug combinations and prediction methods for different molecular disease types in order to enhance the synergistic effect between drugs and reduce the occurrence of side effects [152,153,154].

## 6. Conclusions and Future Perspectives

Personalized medicine is changing the way diseases are diagnosed and treated [155]. The development of modern medicine, especially the advances in drug discovery and development based on new technologies and genomics, provides a solid foundation for personalized treatment. With the continuous advances in science and technology, personalized drug matching, combination therapy, and natural product-based therapy have gradually entered the practice and provide better treatment options for cancer patients. Advances in genomics allow us to better understand the genetic basis of disease and identify individuals most likely to benefit from specific treatments. Pharmacogenomics has proven essential for developing personalized treatment options for patients [6]. By identifying genetic variants that affect drug metabolism, efficacy, and toxicity, clinicians can tailor drug therapy to each patient’s unique genetic makeup, thereby improving clinical care, reducing adverse drug events, and optimizing drug dosing and selection. The development of new technologies has provided the basis for deeper evaluation of genomic data and identification of new therapeutic targets [156]. The development of new technologies and a better understanding of the underlying biology of the disease have enabled the development of more effective targeted therapies [157]. Currently, some chemotherapeutic agents, such as pembrolizumab and nivolumab, are used for the individualized treatment of tumors. Meanwhile, studies have shown that natural products, such as vinblastine, have antitumor effects, in addition to synthetic drugs [158]. Previous studies have confirmed that some natural products are related to pharmacogenomics, which can affect enzymes and transporters of drug metabolism. It is suggested that natural products and traditional Chinese medicine may be potential sources for personalized treatment [159].

In general, the development of personalized therapy has far-reaching prospects, but the challenges cannot be ignored. In the future, we must not only continue to advance research, but also strengthen collaboration and holistic thinking in various aspects. In this way, we can better put personalized treatment into practice and improve patients’ quality of life and the effect of treatment. The integration of pharmacogenomics and other cutting-edge technologies is providing new insights into disease mechanisms and leading to the development of personalized therapeutic strategies. As our understanding of the genetic and molecular mechanisms of disease increases, further breakthroughs in personalized medicine can be expected. These include, in particular, the identification of new drug targets and the development of more effective therapies based on natural products and traditional medicine.

## Figures and Tables

**Table 2:** FDA approved personalized anti-cancer drugs.

Seq_ID	Medicine	Personalized Tag	Approval Time	Molecular Formula	Mechanism of Action	Disease
1	Crizotinib	ALK^+^	2011	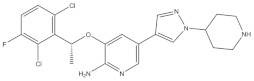	ALK inhibitor	Metastatic non-small-cell lung cancer with ALK or ROS1 positive
2	Ceritinib	ALK^+^	2014	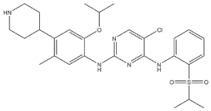	ALK inhibitor	Non-small-cell lung cancer
3	Alectinib	ALK^+^	2015	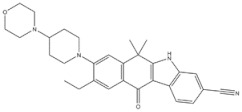	ALK inhibitor	Non-small-cell lung cancer
4	Brigatinib	ALK^+^	2017	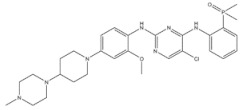	ALK inhibitor	Non-small-cell lung cancer
5	Lorlatinib	ALK^+^ is positive	2018	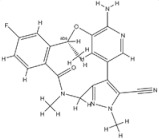	A dual-target inhibitor of ALK/ROS1	Non-small-cell lung cancer
6	Gefitinib	EGFR	2003	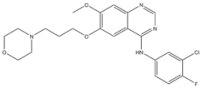	EGFR inhibitor	Non-small-cell lung cancer
7	Erlotinib	EGFR	2004	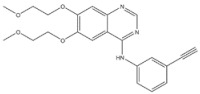	EGFR inhibitor	Non-small-cell lung cancer
8	Afatinib	EGFR	2013	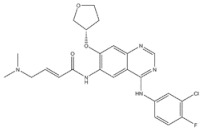	EGFR inhibitor	Non-small-cell lung cancer
9	Osimertinib	EGFR	2015	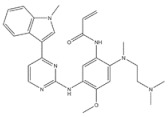	EGFR inhibitor	Non-small-cell lung cancer
10	Pembrolizumab	PD-1	2015	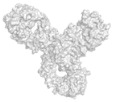 *C_6534_H_10004_N_1716_O_2036_S_46_* *_(PDB:5dk3)_*	PD-1 inhibitor	Non-small-cell lung cancer
11	Nivolumab	PD-1	2014	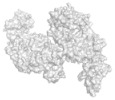 C_6362_H_9862_N_1712_O_1995_S_42_ *_(PDB:5ggr)_*	PD-1 inhibitor	Non-small-cell lung cancer
12	Olaparib	PARP	2014	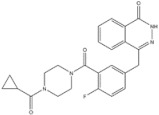	PARP inhibitor	Ovarian cancer
13	Rucaparib	PARP	2016	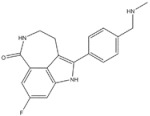	PARP inhibitor	Ovarian cancer
14	Palbociclib	CDK 4/6 kinase	2015	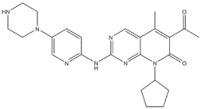	CDK 4/6 kinase inhibitor	Breast cancer
15	Trastuzumab Deruxtecan	HER-2	2022	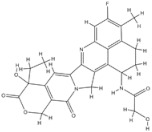	HER-2 inhibitor	Non-small-cell lung cancer
16	Tucatinib	HER-2	2020	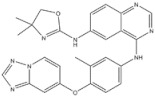	HER-2 inhibitor	Colorectal cancer
17	Vemurafenib	BRAF	2011	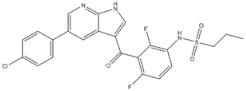	BRAF inhibitor	Metastatic melanoma
18	Larotrectinib	Tyrosinase kinase	2018	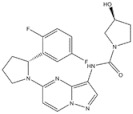	Tyrosinase kinase inhibitor	Solid Tumors
19	Ibrutinib	Tyrosinase kinase	2013	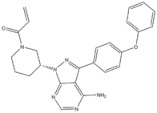	Tyrosinase kinase inhibitor	Mixed lineage leukemia

**Table 3:** Pharmacogenetics in the pharmacological targets and pathways of natural products.

Seq_ID	Natural Products	Main Sources	Molecular Formula	Related Gene	Disease
1	Trabectedin	*Ecteinascidia turbinata*	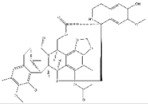	BRCA1, BRCA2	Soft tissue sarcoma, Breast cancer
2	Vincristine	*Catharanthus roseus*	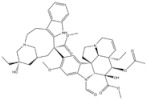	CYP3A enzymes, ABC transporters	Leukemias, Lymphomas, Brain tumors, Solid tumors
5	Paclitaxel	*Taxus baccata Linn*	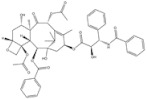	ABCB1 G2677T/A mutation	Ovarian cancer
3	Gigantol	*Dendrobium draconis*	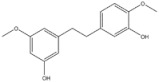	CD133, ALDH1A1	Non-small-cell lung cancer
6	Chrysotoxine	*Dendrobium pulchellum*	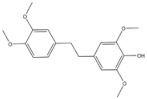	ABCG2	Lung cancer

## Data Availability

Not applicable.

## Abbreviations

EGFR, Epidermal Growth Factor Receptor; ALK, ALK receptor tyrosine kinase; AI, Artificial Intelligence; CADD, Computer-Aided Drug Discovery; MD, Molecular Dynamics; AMBER, Amber Molecular Dynamics; GROMACS, GROningen MAchine for Chemical Simulations; NAMD, NAnoscale Molecular Dynamics; QSAR, Quantitative Structure–activity Relationship; NSCLC, Non-small-Cell Lung Cancer; mCRC, Metastatic Colorectal Cancer; HNSCC, Head and Neck Cancer; GBM, Glioblastoma; GWAS, Genome-wide Association Studies; STS, Soft Tissue Sarcoma; NER, Nucleotide Excision Repair; HRR, Homologous Recombination Repair; CSC, Cancer Stem Cell; PTX, Paclitaxel.
